# Morel-Lavallee Lesion Associated With Rhabdomyolysis in Polytrauma

**DOI:** 10.7759/cureus.63536

**Published:** 2024-06-30

**Authors:** V N K Srinivas Mudiganti, Amol Prakash Singam, Sneha Kakara, Mudiganti Raja Sri Jaya Iswarya, Abhishek Jain

**Affiliations:** 1 Critical Care Medicine, Jawaharlal Nehru Medical College, Datta Meghe Institute of Higher Education and Research, Wardha, IND; 2 Cardiology, Krishna Institute of Medical Sciences (KIMS), Rajahmundry, IND; 3 Radiology, Konaseema Institute of Medical Sciences and Research Foundation, Amalapuram, IND

**Keywords:** morel-lavallee lesion, case report, closed degloving injury, polytrauma, rhabdomyolysis

## Abstract

We describe the case of a 30-year-old man who allegedly had a history of road traffic accidents. While walking on the road, he was hit by a truck. He presented with complaints of facial injuries and being unable to move his right lower limb. On examination, there is a 15x4 cm lacerated wound in the perineal area, with left testes exposed; anal tone could not be assessed; the right lower limb is externally rotated; and deformity is present with palpable peripheral pulses. He was diagnosed with a right sacral ala fracture, a distal one-third shaft of the right tibia fracture, and a right suprapubic rami fracture. Ultrasound of the right thigh showed hematoma and subcutaneous edema all around the gluteal and inguinal regions and fluid collection in the right inguinal region, which is suggestive of Morel-Lavallee lesion (MLL) type 6. On day two of admission, urine was dark in color, and creatinine kinase was elevated, which is suggestive of rhabdomyolysis. He was managed with hydration, electrolyte correction for rhabdomyolysis, and wound debridement for MLL apart from perineal injury, right sacral ala fracture, right suprapubic rami fracture, and distal one-third shaft of the right tibia fracture, with perineal repair and loop colostomy, pelvic binder, and external fixator, respectively. Early identification of the MLL associated with rhabdomyolysis in this polytrauma patient led to recovery and a successful outcome.

## Introduction

Trauma is the leading cause of morbidity and mortality worldwide [1]. It presents challenges in diagnosis and management. Among all the traumatic injuries, two main complications are Morel-Lavallee lesions (MLL) and rhabdomyolysis [1]. MLL can be defined as the separation of skin and superficial fascia from deeper fascial layers, which causes a potential space and leads to fluid accumulation following trauma, particularly in degloving injuries [2]. MLL, first described by French physician Maurice Morel-Lavallee in 1853, is a post-traumatic soft tissue condition characterized by a closed degloving injury. This injury occurs when shear forces separate the skin and subcutaneous tissue from the underlying fascia, creating a potential space filled with hemolymphatic fluid [3]. These lesions are most commonly associated with high-energy trauma, such as motor vehicle accidents, crush injuries, or falls, and are often present in the hip, thigh, and pelvic regions [4]. Clinically, MLL can be challenging to diagnose due to its variable presentation, ranging from an acute, painful swelling to a chronic, asymptomatic mass. Understanding the pathophysiology, diagnostic modalities, and management options for these lesions is crucial for preventing complications such as infection, skin necrosis, and chronic seroma formation [4].

Rhabdomyolysis is a potentially life-threatening medical condition characterized by the rapid breakdown of skeletal muscle tissue, resulting in the release of muscle cell contents, including myoglobin, into the bloodstream. This pathological process can lead to severe complications, such as acute kidney injury (AKI), due to the nephrotoxic effects of myoglobin and other intracellular proteins. The etiology of rhabdomyolysis is diverse, encompassing traumatic causes such as crush injuries and strenuous exercise as well as non-traumatic factors like drug and alcohol abuse, infections, genetic disorders, and metabolic abnormalities [5,6]. Clinically, rhabdomyolysis presents with a triad of symptoms: muscle pain, weakness, and dark-colored urine. However, the severity and presentation can vary widely, ranging from asymptomatic cases to severe systemic manifestations requiring urgent medical intervention. Diagnosis is typically confirmed through laboratory tests showing elevated levels of creatine kinase (CK), myoglobin, and other muscle enzymes, along with renal function tests [7]. The management of rhabdomyolysis focuses on early recognition, aggressive fluid resuscitation to prevent renal failure, and addressing the underlying cause. In severe cases, additional interventions such as dialysis may be necessary [8]. Here, we present a case of polytrauma leading to MLL and rhabdomyolysis.

## Case presentation

**Figure 1:**
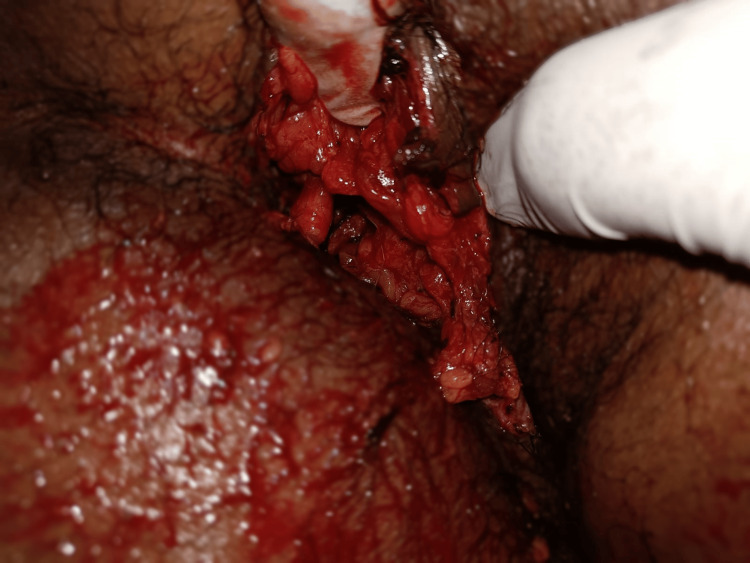
Perineal injury A 15x4 cm perineal lacerated wound in the perineum.

A 30-year-old male patient presented to casualty after a road traffic accident. He was hit by a truck from the back while crossing the road. On presentation, he complained of facial injuries and an inability to move his right lower limb. On examination, there was a 15x4 cm perineal lacerated wound (Figure 1), with the left testes exposed. The anal tone could not be assessed. His right lower limb was externally rotated with palpable peripheral pulses and deformity.

The immediate primary trauma assessment indicated a pulse rate of 120 beats per minute (bpm), blood pressure of 84/60 mmHg, and a delayed capillary refill time. The patient was responsive and given INJ Tetanus toxoid, secured with an IV cannula, and administered a bolus of 1 L of 0.9% normal saline. Laboratory investigations were initiated, including blood grouping, complete blood count, serum electrolytes, creatine phosphokinase, liver and renal function tests, and viral markers.

**Figure 2:**
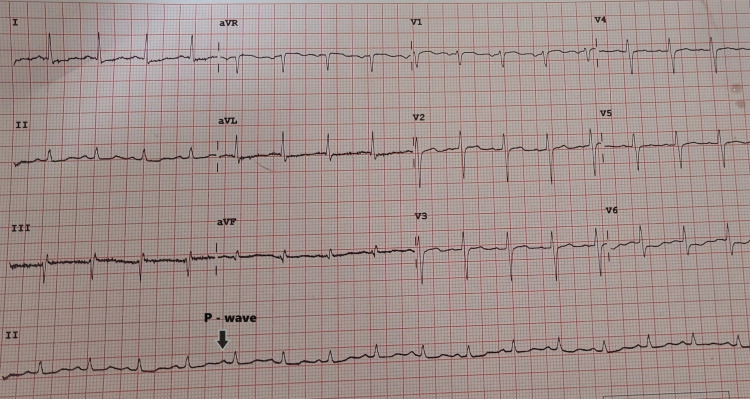
Sinus tachycardia in electrocardiography 1) ECG showing sinus tachycardia with a paper speed of 25 mm/s; 2) regular rhythm with a ventricular rate of more than 100 beats per minute; 3) P-wave with constant morphology preceding every QRS complex.

**Figure 3:**
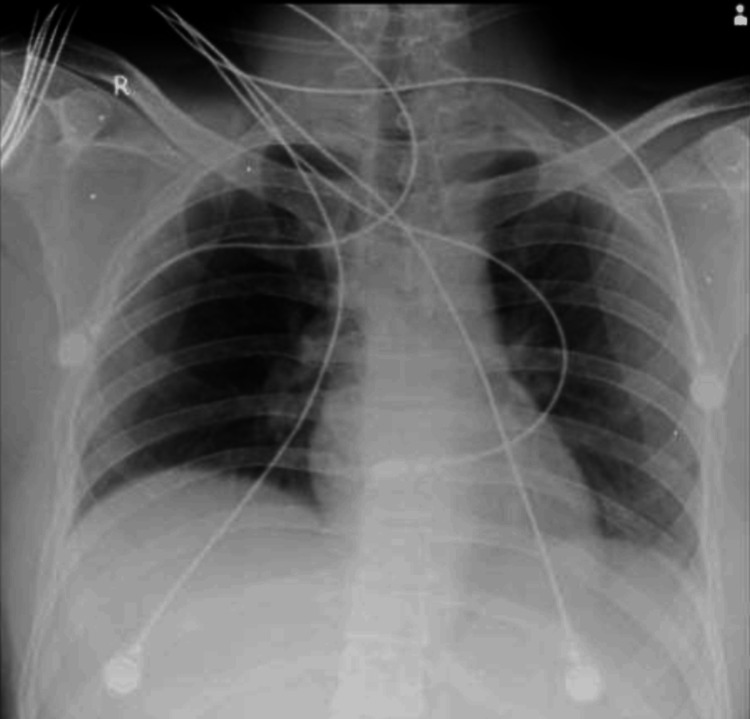
Chest X-ray showing clear lung fields

Further examination revealed abrasions on the forehead, lumbar region, and left thigh, along with open wounds in the bilateral inguinal creases. The Glasgow Coma Scale was 15/15, with equal and reactive pupils. Electrocardiography indicated sinus tachycardia (Figure 2) showing regular rhythm with a ventricular rate of more than 100 bpm. There is a P-wave with constant morphology preceding every QRS complex in the ECG, while a chest X-ray (Figure 3) with clear lung fields. Pneumothorax and hemothorax were ruled out based on chest X-ray.

**Figure 4:**
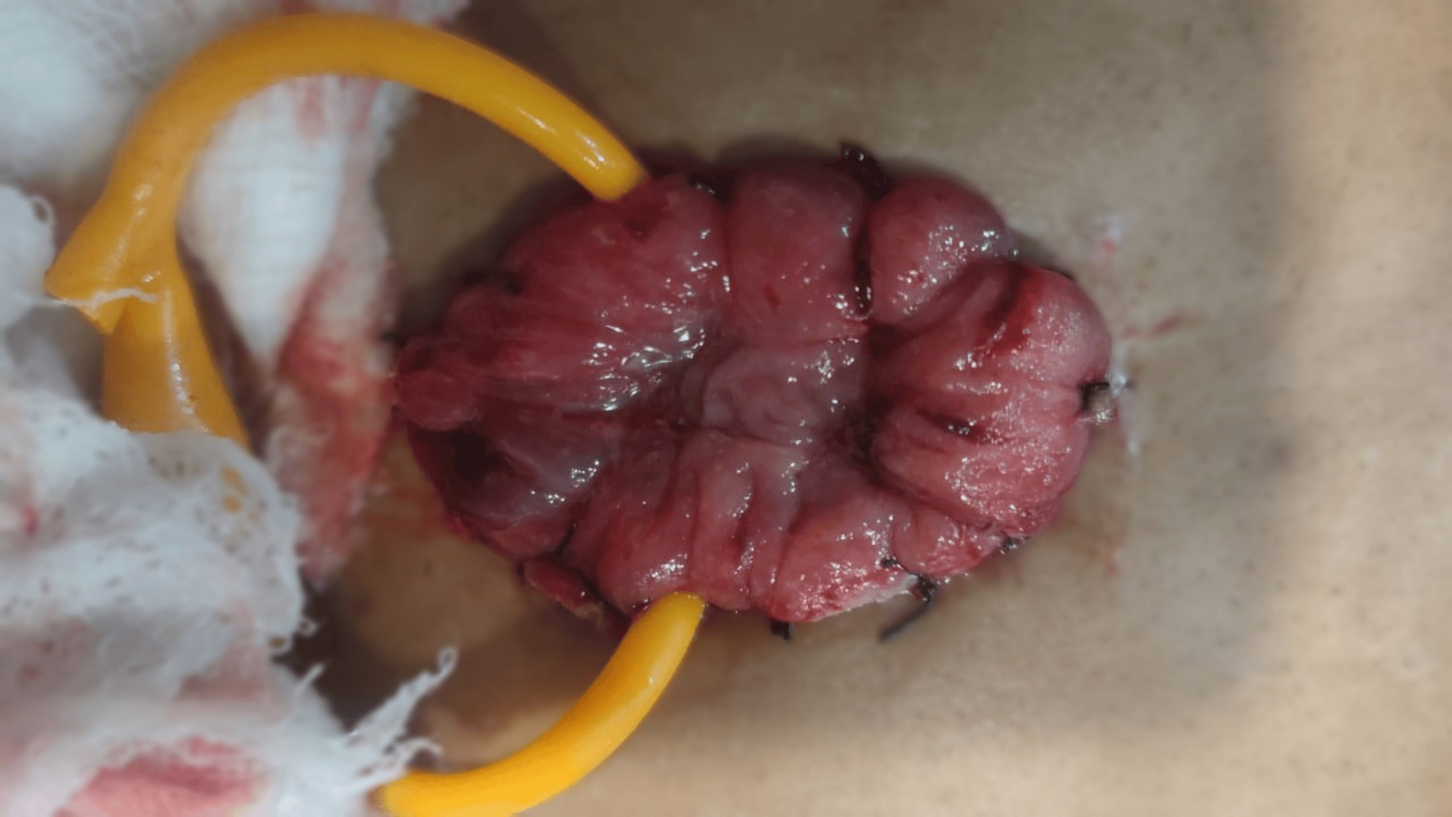
Loop colostomy It is a stoma in which the entire loop of the colon is exteriorized, and both the proximal and distal limbs open into the common stoma opening and are not transected. Loop colostomies are usually temporary and preferred when the colostomy is intended to be reversed at a later date.

**Figure 5:**
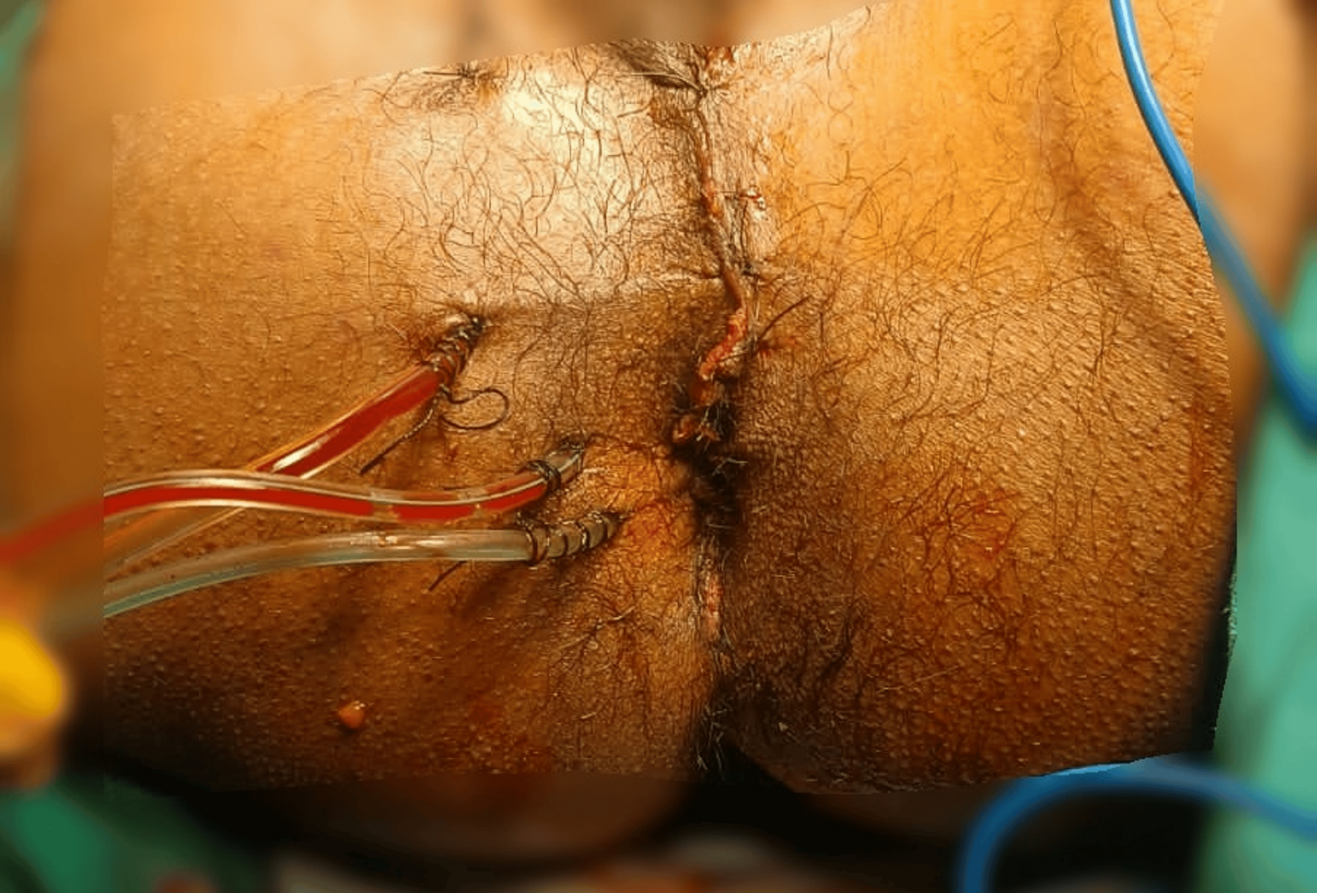
Perineal injury repair Drains in situ.

The extended focused assessment with sonography in trauma (E-FAST) scan did not show any signs of pericardial effusion, free fluid in the hepatorenal interspace, splenorenal interface, or peritoneal space, and pneumothorax was ruled out. A Foley catheter was inserted, and loop colostomy (Figure 4), perineal injury repair (Figure 5), and external fixator placement were performed in the same sitting due to polytrauma.

**Figure 6:**
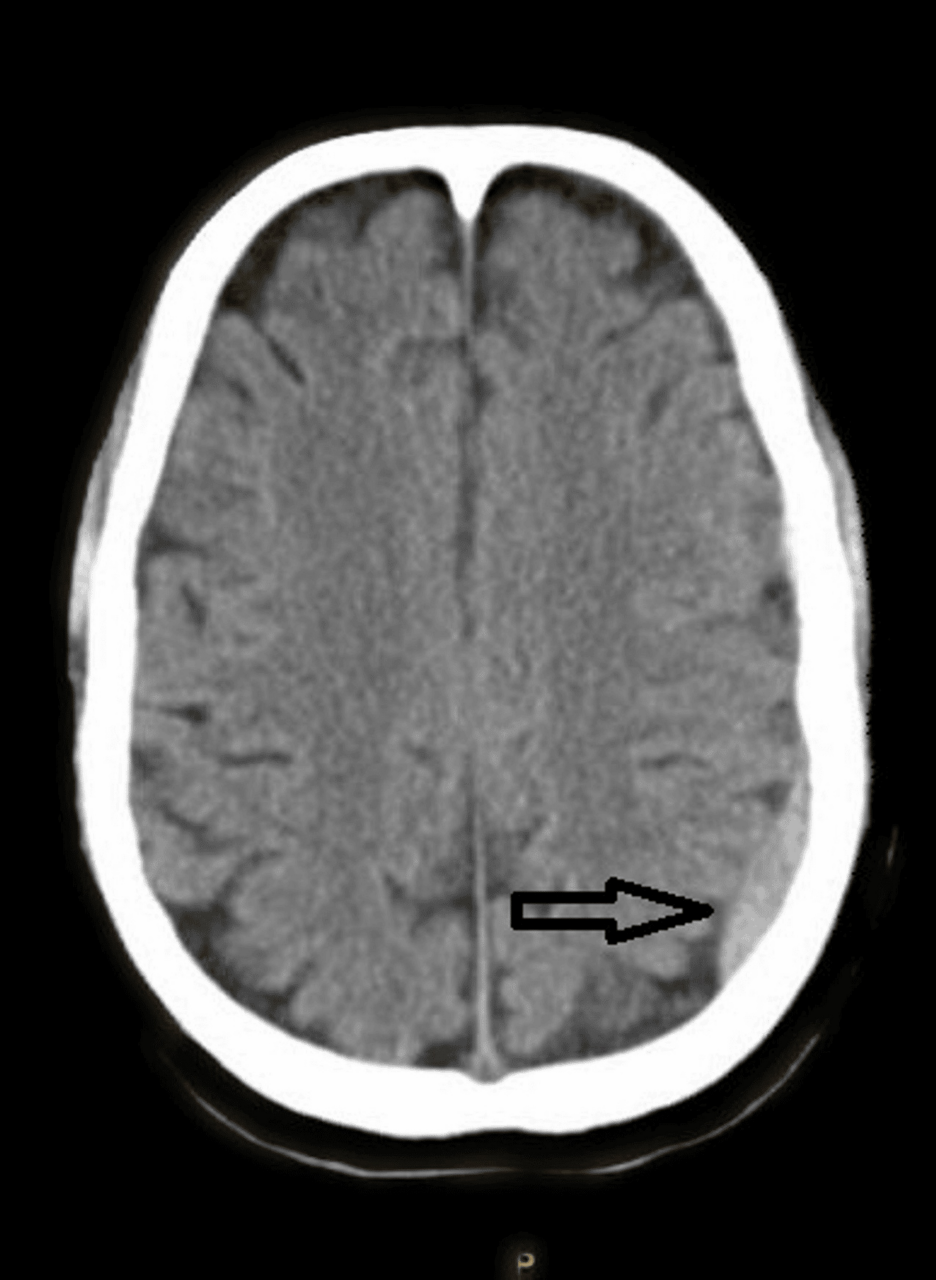
Non-contrast computed tomography of the brain showing a left parieto-occipital subdural hematoma

As per hospital protocol, computed tomography of the brain revealed a left parieto-occipital subdural hematoma (Figure 6) without any skull fracture.

Postoperatively, the patient was transferred to the critical care unit, where a central venous catheter was placed, and treatment commenced with injection piperacillin and tazobactam 4.5 g intravenously stat dose followed by three times a day, injection metronidazole 500 mg IV TDS, injection levetiracetam 500 mg BD, intravenous fluids based on the urine output, and fentanyl infusion at 2 mcg/kg/hour. Two pints of packed red blood cells were administered intraoperatively and postoperatively, and chest physiotherapy was initiated. Deep vein thrombosis (DVT) prophylaxis was provided with a compressible stocking device.

Postoperative days (POD) 1-6 progression

POD 1

The patient remained conscious and responsive, with a GCS score of E4V5M6. Vital signs included a blood pressure of 110/70 mmHg, heart rate of 104/minute, and oxygen saturation of 99% on room air. The laboratory results are presented in Table 1. Additionally, the fluid input and urine output were adequate.

**Table 1:** Laboratory investigations An increase in creatine kinase is suggestive of rhabdomyolysis. POD: postoperative day

Investigations	POD 1	POD 2	POD 3	POD 4	POD 5	POD 6	Normal Value
Hemoglobin	8.5	8.1	10.2	9.1	8.8	8	14-18 g/dL
Total white blood cell count	10800	5700	17400	27100	17900	17600	4500-11000/µL
Total platelet count	1.6 lakh	2.84 lakh	3.43 lakh	4.36	5.94	4.83	1.5-4.5 lakhs/µL
Urea	17	45	65	52	54	38	5-20 mg/dL
Creatinine	0.6	1.1	1.9	1.6	1.1	1	0.7-1.3 mg/dL
Sodium	139	138	133	126	124	136	135-145 mEq/L
Potassium	4.5	3.4	5.4	5.2	4.9	4	3.5-5.2 mmol/L
Creatine kinase	-	3200	5137	4858	2485	1121	45-250 U/L

POD 2

**Figure 7:**
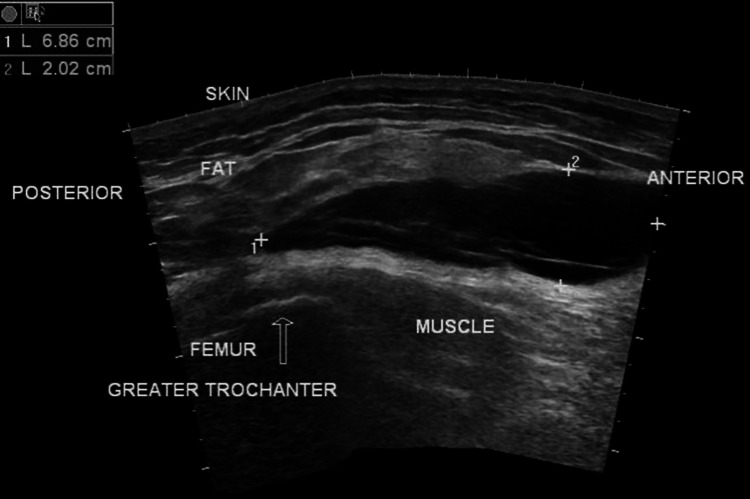
Ultrasound finding of the Morel-Lavallee lesion Ultrasound image showing a 6.86 cm × 2.02 cm accumulation under the skin and fat layer and above the muscle near the greater trochanter of the femur.

The patient's consciousness and vital signs were stable, with a slight increase in heart rate and body temperature. Ultrasound of the right thigh revealed inflammatory changes consistent with an MLL type 6 (Figure 7).

These lesions are anechoic or hypoechoic. Hematoma can be predominantly echogenic in the acute phase, becoming more hypoechoic as blood products liquefy over time. Conservative management was initiated with magnesium sulfate dressing.

POD 3

The patient developed dark-colored urine indicative of rhabdomyolysis, confirmed by elevated CK levels. Adequate hydration was ensured, avoiding solutions with high potassium content due to subdural hematoma. Fever ensued, and samples were collected for culture and sensitivity testing.

POD 4

**Figure 8:**
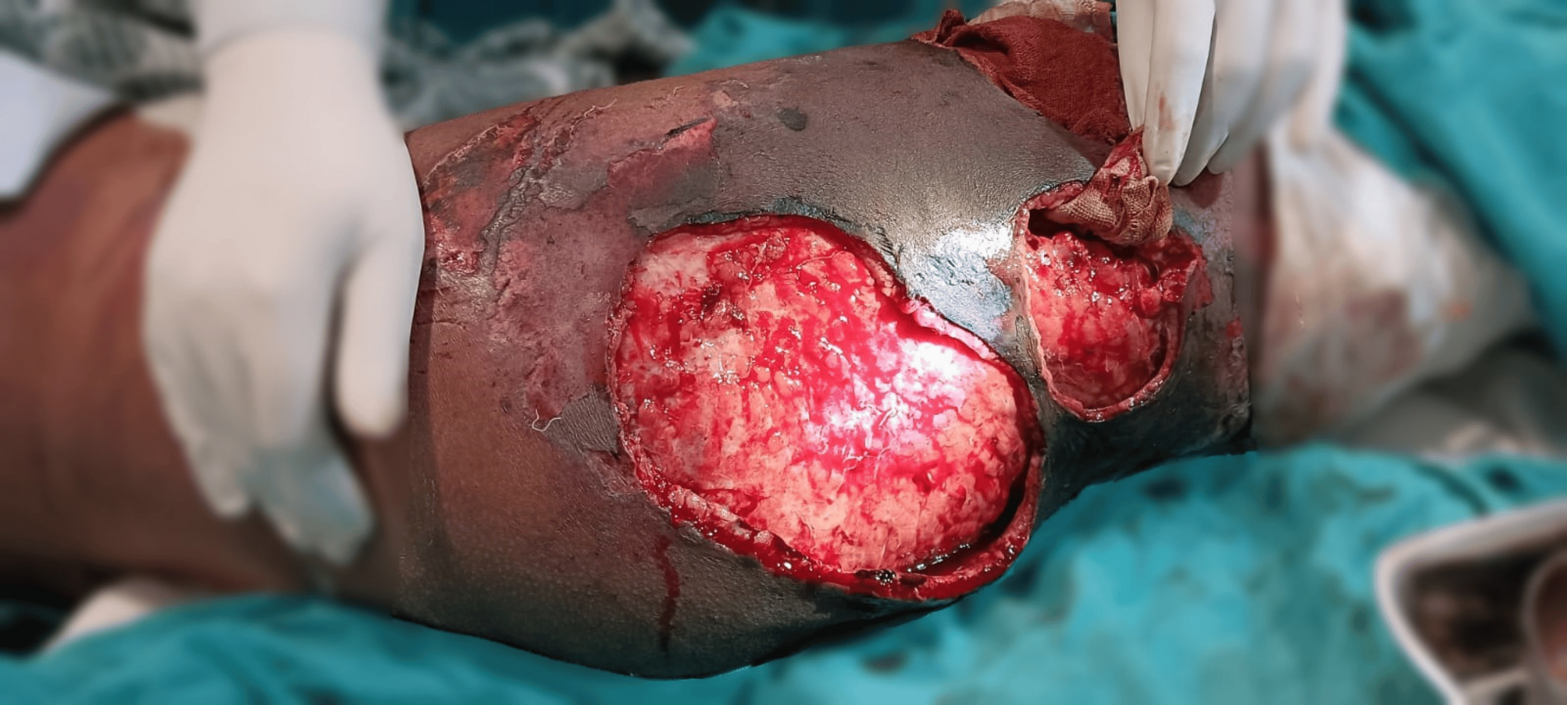
Wound debridement on the thigh Wound debridement was performed after diagnosing the patient with a Morel-Lavallee lesion.

Despite the correction of hyponatremia, the patient exhibited tachycardia, tachypnea, and metabolic acidosis. Wound debridement (Figure 8) was performed at the bedside, and electrolyte imbalances were addressed. Further investigations and antibiotic therapy continued.

POD 6

The patient's condition deteriorated, manifesting with fever, leukocytosis, and extensive cuticular necrosis on the thigh. Electrolyte abnormality was present for which all required corrections were given. Extensive wound debridement was done. The blood culture and sensitivity report showed the growth of coagulase-negative staphylococci sensitive to the Penicillin group of drugs. Therefore, the patient's treatment was maintained with the same medications. DVT prophylaxis was given. The patient was discharged on request.

## Discussion

In this case, a trauma survey is important in identifying life-threatening injuries and guiding inappropriate intervention [9]. Imaging modalities such as computed tomography and ultrasonography [10] play a main role in identifying internal injuries such as subdural hematoma and MLL [11]. Early hemorrhage control, fluid resuscitation, and limb stabilization were important in preventing further deterioration and stabilizing the patient for surgical interventions. Timely surgical interventions, such as the loop colostomy and external fixator placement, addressed immediate complications and prevented further damage.

Rhabdomyolysis was identified by a fivefold elevation of serum CK levels and dark-colored urine. Timely identification leads to proper management through fluid therapy and the further prevention of renal failure. Coordination between trauma surgeons, critical care doctors, infectious disease specialists, and rehabilitation specialists was necessary for the patient's care. This multidisciplinary approach made certain that all issues involving the patient were addressed. Modern monitoring methods, such as lung ultrasonography for determining hydration status and bedside lung ultrasound in emergency (BLUE) protocol-based B-line detection, have demonstrated how crucial sophisticated monitoring instruments are for directing fluid management and averting problems. The importance of early identification and management of complications such as MLL and rhabdomyolysis in polytrauma cannot be overstated. Timely interventions can significantly improve outcomes and prevent long-term sequelae. Effective trauma care requires coordination among various specialties, including trauma surgery, critical care, infectious disease management, and rehabilitation services. This collaborative approach ensures comprehensive care and addresses all aspects of the patient’s injuries and recovery needs. Trauma care is dynamic, and continuous monitoring is essential to adapt treatment strategies based on the evolving clinical picture. Advanced monitoring tools and protocols play a crucial role in guiding management decisions [12].

In a case report of Ketan Vagholkar, magnetic resonance imaging (MRI) was done to confirm the diagnosis. A limited open approach for evacuating the fluid in the lesion was performed, followed by irrigation of the cavity with a combination of 3% hypertonic saline and hydrogen peroxide to induce fibrosis and obliterate the dead space. This was followed by continuous negative suction accompanied by a pressure bandage [13].

In another case reported by Durrani et al., bedside ultrasonography revealed a large anechoic fluid collection in the deep subcutaneous plane with mobile internal echogenic debris. Later, the patient underwent contrast-enhanced CT of the affected lower extremity, demonstrating a fluid collection superficial to the deep fascia of the distal posteromedial left femur, confirming the diagnosis of an MLL [14].

In our case report, as it is a rural hospital with a limited resource setting, we stick to clinical exams and ultrasound findings to confirm the diagnosis of MLL.

The patient's choice to leave the hospital against the recommendation of the doctors emphasizes the necessity of providing comprehensive patient education regarding the dangers and significance of follow-up treatment. For the best results, patients and their families must comprehend the consequences of their injuries and the need for further care.

## Conclusions

In conclusion, polytrauma cases need to be evaluated thoroughly on arrival by primary and secondary surveys, and a tertiary trauma survey should be conducted at the intensive care unit. This can give a better picture of the patient's condition, and treatment modalities can be planned accordingly. The association of polytrauma with Moral-Lavalle lesions and rhabdomyolysis highlights the need for heightened awareness and proactive treatment approaches. Ensuring timely and thorough medical intervention can significantly improve patient outcomes, mitigating the risks associated with these potentially severe conditions. In our case, the early diagnosis and good interdepartmental coordination improved patient outcomes.
